# Cortical Vision Impairment (CVI)-informed assessment and treatment of challenging behavior in a child with SCN2A-related disorder

**DOI:** 10.1186/s11689-024-09580-7

**Published:** 2024-11-28

**Authors:** Benjamin R. Thomas, Natasha N. Ludwig, Danielle Pelletier, Melanie Bauer, Rebecca Hommer, Constance Smith-Hicks, Julia T. O’Connor

**Affiliations:** 1 Department of Behavioral Psychology, Kennedy Krieger Institute, Baltimore, MD USA; 2 Present Address: Center for Autism Spectrum Disorders, Nationwide Children’s Hospital, 189 West Schrock Rd, Westerville, OH 43081 USA; 3 Department of Neuropsychology, Kennedy Krieger Institute, Baltimore, MD USA; 4 Connections Beyond Sight and Sound, University of Maryland, College Park, MD USA; 5 Department of Neurology and Developmental Medicine, Kennedy Krieger Institute, Baltimore, MD USA; 6 Johns Hopkins University School of Medicine, Baltimore, MD USA

**Keywords:** Autism spectrum disorder, Cortical visual impairment, Applied behavior analysis, Functional behavior assessment, Functional vision assessment, Parent training, SCN2A-related disorder

## Abstract

This report presents results of parent-implemented behavioral treatments for a child with cortical visual impairment (CVI), intellectual disability (ID), epilepsy, and autism spectrum disorder (ASD) associated with a pathogenic variant in the SCN2A gene (i.e., SCN2A-Related Disorder). Treatment evaluations were informed by combined results of functional behavior assessment (FBA) and functional vision assessment (FVA) which yielded CVI-related accommodations. The treatment of escape-maintained challenging behavior involved the evaluation of behavioral prompting strategies in accordance with CVI-related accommodations, extinction (EXT), and differential reinforcement modifications. The treatment for behavior problems maintained by access to food (tangible-edible) included functional communication training (FCT), EXT, and schedule thinning with schedule-correlated visual signals. Overall, integrating child-specific CVI-related accommodations was essential for developing effective behavioral interventions for this child. FVAs are accessible and practical for uptake by behavior analysts in vision-informed assessment and treatment of challenging behavior.

Cortical visual impairment (CVI) is the most common type of visual impairment in western countries and originates in the posterior visual pathway rather than the eye or optic nerve [8, 19]. CVI is associated with disturbances in processing visual information (e.g., perception, attention, sensitivity to complexity) and presents with a constellation of characteristic visual behaviors (e.g., preference for bright colors, photophobia, side viewing [11, 35]. CVI can range from mild to severe and requires accommodations to support visual processing. Furthermore, the types of accommodations needed can vary based on the child. CVI is most associated with perinatal brain injury but is frequent in other causes of congenital or acquired neural dysfunction such as hydrocephalus, epilepsy, and neurogenetic conditions (Boonstra et al., 2002, [19]). Other ophthalmologic abnormalities may also be observed in children with CVI, including esotropia, exotropria, nystagmus, and optic atrophy [19].

CVI is common in children with neurodevelopmental disorders (NDDs), which can make differential diagnosis and treatment of CVI-related challenges more difficult, given risk of diagnostic overshadowing (Chockron & Dutton, 2023; [18],Ludwig et al., 2021). For instance, there is a degree of overlap in the behavioral presentation of autism spectrum disorders (ASD) and children with visual impairment, such as motor stereotypies and impaired social interactions (i.e., “blindisms”; [18]). Challenges encountered in assessment and treatment may also be relevant to consider for children with vision impairment and NDDs who are shown to have a heightened risk for externalizing behavioral problems [1]. For instance, characteristics of CVI can be misinterpreted as low attention, skill deficit, or defiance [35]. Additionally, challenges in communication and adaptive skills that are often associated with NDDs and vision impairment can increase risk for behavior problems [4, 30]. Research also indicates that several organic and environmental factors, as well as the extent of the child’s visual disturbance, may differentially contribute to the likelihood of behavior problems [11, 31]. Combined, these factors may mask the need for vision assessment and preclude the provision of adequate behavioral supports.

Given the heterogeneity of visual disturbances associated with CVI, a multidisciplinary team approach is often recommended to address the diverse challenges encountered in assessment, education, and treatment [8], Ludwig et al., 2021; [35]. Professional coordination may also benefit in treating externalizing behavior problems in children with CVI and NDDs. This paper presents a case of multidisciplinary treatment of behavior problems in a child diagnosed with CVI, intellectual disability, and ASD associated with a pathogenic variant in the SCN2A gene (i.e., SCN2A Related-Disorder). Behavioral treatment was guided by the results of a) a functional behavior assessment (FBA) with functional analysis (FA) that identified environmental factors influencing behavioral outbursts (i.e., antecedents and consequences), and b) a functional vision assessment (FVA) that evaluated the individual’s functional vision through assessment of unique vision behaviors and challenges associated with CVI across routine tasks and activities, and provided a unique set of CVI-related accommodations and supports [27].

## Method

### Participant, settings, and materials

The patient, Adam, was a 6-year-old male with ASD, epilepsy, and intellectual disability secondary to a likely pathogenic variant c.3399G > C in the SCN2A gene that confers a diagnosis of SCN2A-RD. Adam was initially screened in a synaptopathies multidisciplinary clinic within a university-based medical center, and then subsequently referred to the clinic’s outpatient program for the treatment of severe challenging behavior. His full demographics are presented in Table 1. Briefly, pathogenic variants in the SCN2A gene affect the function of voltage-gated sodium channels (NaV1.2) in excitatory cortical and subcortical neurons [5, 32]. SCN2A-RD is one of the most common monogenic causes of ASD [29]. Adam’s history is notable for having a diagnosis of cortical visual impairment (CVI). Emerging research shows that vision problems are reported in approximately 68% of patients with SCN2A-RD (e.g., visuomotor, dept, and/or distance problems), including CVI occurring in upwards of 42% of patients [6, 7].

**Table 1:** Patient demographics

**Sex at birth**	**Age (y)**	**Ethnicity**	**Family Hx**			
Male	6	White	No			
**Gene**	**Variant**	**Classification**	**Zygosity**	**Chr. Position**	**Mode of Inheritance**	**Inherited** **From?**
SCN2A	c.3399 G>C p.E1133D	Likely pathogenic	Heterozygous	Chr2:g.166211181G>C	AD	De Novo
**Medical diagnoses**		**Psychiatric diagnoses**		**Medications**		
Cortical vision impairment (CVI), partial symptomatic epilepsy with complex partial seizures, sleep apnea, periodic limb movement disorder		Autism spectrum disorder (ASD), intellectual disability, adjustment disorder with other symptoms, mixed receptive-expressive language disorder, disruptive behavior disorder		Diazepam, levetiracetam, melatonin, pediatric multivitamin, valproate		
Developmental level*						
*Cognitive: *14 month-level						
*Receptive Communication: *10 month-level						
*Expressive Communication: *7 month-level (no spoken language)						
*Fine Motor: *14 month-level						
**Aberrant Behavior Checklist-Community (ABC-C **[2]**)**						
*Irritability: *11 of 45 MSP						
*Lethargy/Social withdrawal*:12 of 48 MSP						
*Stereotypic behavior: *14 of 21 MSP						
*Hyperactivity/noncompliance*: 23 of 48 MSP						
*Inappropriate speech:* 2 of 12 MSP						

Note: *Bayley Scales of Infant and Toddler Development, Fourth Edition. This was administered out of the standardized age-range of the Bayley-4, so standardized scores are not available. *MSP *Maximum score possible

Adam was reported to request by tapping on things he wanted, and he had some prior success signing "more” as a general request. His current speech program was teaching a picture-based communication system with limited success, and he did not have an appropriate modality to dissent/reject. Adam had almost no independence in daily routines as parents placed few demands on him at home to avoid self-injury. At the time of intake, he attended a center-based Applied Behavior Analysis (ABA) program for 40 h/week and was also followed by a sleep clinic.

The behavioral intervention described here, involved 2-h appointments, twice per week, for 8 weeks. Approximately 5–10 assessment or treatment sessions occurred per appointment. His biological mother participated in his assessments and treatments. She was a college-educated professional with no prior experience or training in behavioral treatment. Functional Analysis (FA) and initial treatments occurred in a 9 × 11’ treatment room in an outpatient clinic. Treatment extension sessions occurred in various rooms in the clinic and Adam’s home via telehealth. Tangible treatment materials consisted of Adam’s preferred snack foods, a laminated 2 × 2″ icon depicting “snacks,” and an 8.5 × 11″ laminated board, that was evenly partitioned on the top with the colors red and green to signal the availability/unavailability of snacks. Initial demand materials included colored blocks for matching, a ring stacker, a bin for putting toys away, and a box for elevating task materials. Extensions included home tasks of packing book bag, dressing, cleaning up toys/room, etc.

### Measurement, Interobserver Agreement (IOA), and procedural integrity

#### Measures

There were three dependent variables. *Challenging behavior* was defined as any instance of self-injury (hand to head or hand to back hitting), aggression (hitting, pinching, or biting others), or disruption (hitting or kicking objects/surfaces, or throwing objects). *Functional communication response (FCR)* was defined as Adam handing a laminated 2 × 2″ icon to the caregiver to obtain food. Frequency of challenging behavior and FCR were divided by session time to reflect a response per minute (rpm). *Cooperation* involved emitting a correct response within the designated time frame, with independence or following a gesture or model prompt. Percentage cooperation was calculated by summing the total number of correct responses, by the total number demands in a session and multiplying the quotient by 100.

#### IOA

Data were collected using laptop computers and behavioral data software (BDataPro; [9]). A trained secondary observer collected data for 51% of Adam’s tangible-edible and 43.6% of his demand treatment sessions (including FA baseline). IOA was calculated with an exact interval agreement method. For the tangible-edible treatment, IOA was 97.3% for challenging behavior and 97.3% for FCR. In the demand treatment, IOA was 98.2% for challenging behavior and 98.8% for cooperation.

#### Procedural integrity

Adam’s mother demonstrated high levels of procedural integrity across FA and baselines (*M* = 83.4%), demand treatment evaluation (*M* = 94.3%), and demand extension phases (*M* = 96.4%). This level was also maintained in the tangible treatment evaluation (*M* = 94.5%), including extensions (*M* = 100%).

### Procedure

#### Functional Behavior Assessment (FBA)

At intake, a hierarchy of Adam’s preferred items for use in assessment and treatment were identified with multiple stimulus without replacement preference assessment (MSWO; [12]). Adam was then observed in semi-structured, general situations with his mother to operationalize his challenging behavior, and note caregiver interaction and management strategies (e.g., praise style, common demands, etc.). Scenarios included demands, removing preferred items or food, low attention, and play. Each session was 5 min, and parent was instructed to react how she typically would at home.

Adam then participated in a parent-conducted functional analysis (FA; [17]/94). Staff taught mother the FA procedures to proficiency (i.e., > 90% fidelity) using instructions, modeling, rehearsal, and feedback [20]. During the *toy play* condition of the FA (i.e., control), Adam was provided continuous access to preferred items, positive attention, and no demands were issued. Challenging behavior was ignored. In the *attention* condition, parent provided moderately preferred items, informed Adam she was “busy,” and then pretended to be occupied. Contingent upon challenging behavior, parent issued attention in the form of a mild reprimand (e.g., “Don’t hit yourself!”). The *demand* condition involved academic and daily living demands involving 3-D objects and delivered with least-to-most prompting (e.g., verbal, model, hand-over-hand; [16]). Adam’s cooperation was praised, and challenging behavior resulted in a 30-s break. Adam accessed a highly preferred item for 1 min prior to *tangible* sessions. At the start of session, parent removed and withheld the item. Contingent upon challenging behavior, parent returned the item for 30 s. Finally, the *tangible-edible* condition involved preferred foods in lieu of items. Sessions were 5 min in duration, and all contingencies were repeated until the end of session.

#### Functional Vision Assessment (FVA)

The Cortical Vision Impairment Assessment and Intervention Tool [27] was conducted prior to commencement of treatment by a certified doctoral-level teacher for the visually impaired (BH). The FVA process examines the presence of functional vision as it relates to CVI to inform interventions. The assessment protocol utilizes information gleaned from the referral, parent interview, observation, and direct assessment with the CVI Range Tool. The CVI Range Tool component is utilizes a variety of activities, both child and assessor guided, that are designed to identify obvious and subtle manifestations of CVI. This approach allows the examiner to observe and rate 10 characteristics of CVI including color, movement, latency, visual fields, complexity, light, distance viewing, visual reflexive responses, novelty, and visual motor skills. Results yield a CVI range score, from 0 (no functional vision) to 10 (near typical visual responses). The CVI Range Tool also places a child within one of three phases with Phase 1 being most severe and Phase 3 being least severe. The assessment occurred in Adam’s home during routine activities (e.g., playing with toys, snack, interaction with mother).

#### Treatment evaluation

Reversal designs were used to evaluate Adam’s treatment packages (Bear et al., 1968). Adam’s initial baselines were comprised of data from the previously conducted FA. Reversal to baselines replicated the respective FA condition, and treatment reversals replicated prior treatment phases. Phase change decisions for the escape-maintained challenging behavior treatment involved consideration of reductions in challenging behavior and co-occurring increases in cooperation. Adam’s mother was the primary implementer of treatment. Staff used instructions, modeling, rehearsal, and feedback in training her to proficiency for each treatment (i.e., > 90% fidelity, e.g., [20]). Treatment materials remained in clinic until extended to family home. All treatment sessions were 5 min.

### Tangible-maintained challenging behavior

#### Functional communication training (FCT; pre-treatment)

Functional communication training (FCT) teaches individuals to emit an appropriate communication behavior to access the reinforcers that maintain challenging behavior (e.g., [10]). Adam was taught to exchange a laminated, 2 × 2″ picture icon for snacks, using least to most prompting, with a 5-s prompt delay. At the start of each trial, the caregiver presented Adam with the 2 × 2″ icon and then withheld snacks. Contingent upon Adam handing the icon to his caregiver, regardless of prompt level or display of challenging behavior, he was provided snacks for 30 s. All challenging behaviors were ignored. There were 10 trials per session, and mastery criterion was 3 consecutive sessions with ≥ 80% independent exchanges. Training trials are excluded from the treatment evaluation figure.

#### FCT + extinction (EXT)

Prior to the session, Adam was provided 1 min to consume snacks. At the start of the session, the caregiver held up an 8.5 × 11″ signal board, evenly partitioned with the colors green and red. Per FVA results and CVI accommodation recommendations (e.g., [35]), the red/green provided contrast against other environmental stimuli and holding up the board kept it in his visual field. The caregiver placed the snack icon on the green side to indicate that independently emitting the FCR (card exchange) would be reinforced with snacks (i.e., S^D^). Adam was informed that snacks were “all done,” and if he wanted more snack he should “ask nicely”, and then removed the food. No more prompts were provided. An independent FCR produced access to snacks for 30 s, and then the caregiver removed the snacks again. This sequence was repeated until the session time elapsed. All challenging behavior was ignored or blocked for safety.

#### Reinforcement schedule thinning

Following ≥ 80% reduction in challenging behavior in the second treatment phase (from the baseline average), a multiple schedule of reinforcement was introduced, using additional schedule-correlated visual stimuli (i.e., signaled availability; SA). The purpose of this phase was to make the treatment more practical for his family by teaching Adam adherence to scheduled family meals versus continuous grazing. During a designated period within the session, the caregiver presented the signal board, and affixed the snack icon to the red side of the board to indicate that snacks were unavailable, and the FCR (for snacks) would not be reinforced (i.e., S^∆^). Upon showing the signal, the caregiver announced the rules verbally, and removed his snack. After a predetermined amount of time, along with the absence of challenging behavior and attempts to use FCR during the final 5 s of the interval (i.e., changeover delay), the caregiver placed the icon on the green side of the board and informed Adam that the FCR would again produce snacks (i.e., S^D^). The initial S^∆^ was 30 s, and this was lengthened using a terminal probe approach to thinning according to Strohmeier et al. [33].

### Escape-maintained challenging behavior

#### Differential reinforcement of alternative behavior (DRA) + EXT

A treatment package comprised of differential reinforcement of compliance and extinction was used to address Adam's challenging behavior maintained by escape from demands. At the start of the session, the caregiver seated Adam at a table and initiated demands using a standard three-step prompting approach (e.g., [16]) with a 5-s prompt delay between each step. Contingent upon cooperation with independence or following a gesture/model prompt, the caregiver provided praise (e.g., “Great job Adam!”) and removed demand materials for 30 s. No demands were issued during this time to ensure reinforcement involved escape from demands. After 30 s elapsed, the caregiver resumed demands and repeated this sequence as applicable until the session ended. All challenging behavior was ignored or blocked for safety.

#### DRA + EXT + Enriched break (EB)

During sessions with enriched breaks, Adam’s task cooperation (with independence or following a gesture/model prompt) was reinforced with praise, the removal of demands, along with the addition of preferred items and mother's positive attention for 30 s. Preferred items were identified via MSWO preference assessment [12] prior to sessions, and the top 3 items were provided concurrently during the break.

#### DRA + EXT + Prompt modifications (PM)

This treatment replicated the DRA + EXT except for modifications to the three-step prompting sequence. Modifications were guided by results of the FVA and included: *Position* (presenting materials at or above eye level), *Orienting/Familiarity Cue* (each item was presented individually until he looked at it), *Movement* (items were wiggled during orienting cue to augment salience during orienting), and *Latency* (time delay increased to 20 s between prompts).

#### Extensions

Treatment extensions for the final tangible-edible treatment involved novel therapists (clinic staff in two sessions) and during meals with signal board posted on refrigerator in the kitchen of his home via telehealth. The escape from demands treatment included new demands in various rooms in his home via telehealth (adaptive living skills such as dressing, packing/unpacking backpack).

## Results

### FBA

**Fig. 1:**
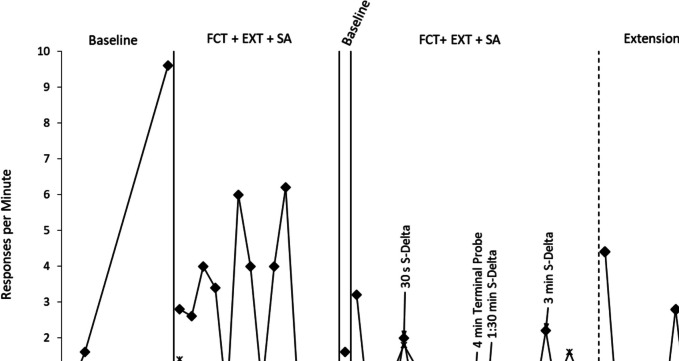
Results of the tangible-edible treatment FCT = Functional Communication Training; EXT = Extinction; SA = Signaled Availability

**Fig. 2:**
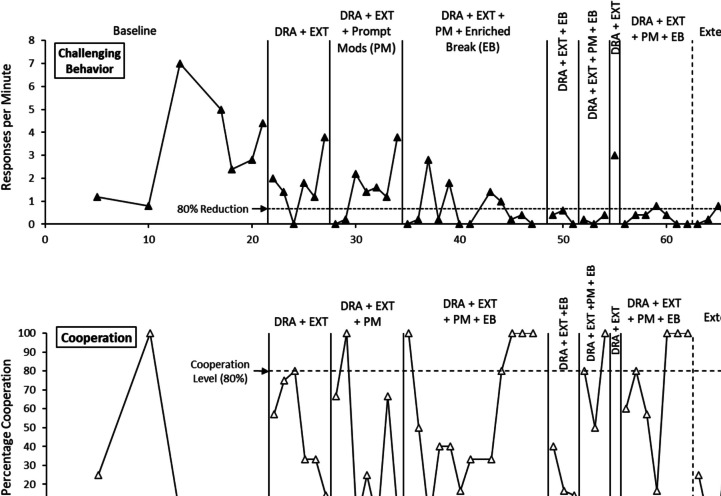
Results of the demands treatment DRA = Differential Reinforcement of Alternative Behavior; EXT = Extinction

Results of the preference assessment indicated snacks and tablet were Adam’s most preferred items, and these may function to reinforce his behavior. Adam’s semi-structured observations were inconclusive, warranting further assessment with FA methods. FA results showed that Adam’s challenging behavior frequently occurred in the tangible-edible condition (*M* = 4.0 rpm; range, 0.8–9.6 rpm) and demand condition (*M* = 3.28 rpm; range, 0.8–5.0 rpm). Additionally, Adam rarely cooperated with expectations within the demand condition (*M* = 17.86%). Low rates of challenging behavior were observed in the toy play, tangible, and attention conditions (range, 0.00–0.80 rpm). Overall, results suggested that Adam's behavior problems occur to access food and to escape demands (see Figs. 1 and 2 for FA baselines).

### FVA

Results of the FVA are presented in Table 2. Adam’s CVI Range score was 5.75–6.25, placing him in Phase 2 CVI. Per the evaluation report, he demonstrated impact across all 10 CVI characteristics. Recommendations detailed how to provide accommodations to promote access to visual stimuli and support the interpretation of visual stimuli. Generally, recommendations guided by the FVA articulated that gaining and sustaining Adam’s attention was best with 3-D objects, object movement, increased time for visual latency, and presentation in central and superior visual fields along with simple and contrasting backgrounds (e.g., 2–3 colors). These components were then incorporated and evaluated in his treatment (see prompt modifications).

**Table 2:**
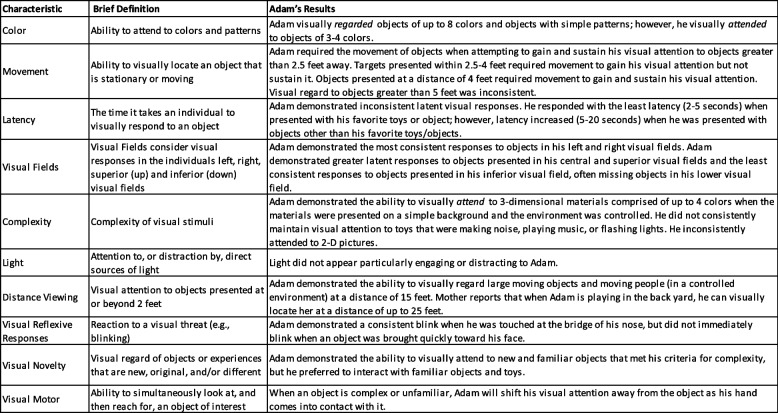
Summary of Results from Adam’s CVI Range Assessment by CVI Characteristic

### Treatment evaluation

#### Tangible-edible maintained challenging behavior

Treatment results for challenging behaviors maintained by access to tangible-edibles are presented in Fig. 1. Adam’s challenging behavior was elevated during the initial FA baseline (*M* = 4.0/min). Adam completed 8 sessions of FCT to meet mastery criterion. During FCT + EXT, Adam’s challenging behavior decreased to an average of 2.47/min across 14 sessions, with low rates observed in the final 4 sessions after the changeover delay was added (range, 0.0–0.20/min). Challenging behavior increased upon reversal to baseline (*M* = 1.60/min), and then decreased when FCT + EXT was reinstated (*M* = 0.90/min), representing a 77.5% reduction from FA baseline. Across the sessions, Adam’s FCR averaged 0.86/min, and 0.90/min, respectively. Next, the availability of reinforcement was systematically thinned in the S^∆^, from 30 s to 3 min. Challenging behavior remained minimal, with an average of 0.15/min observed in the final 4 sessions (96.3% reduction from FA baseline). Adam’s FCR varied, depending on availability (*M* = 0.82/min). During the extension phase, Adam’s challenging behavior maintained at zero except for an initial increase with novel people (2 sessions), and during one session in his home (via telehealth). The final sessions involved a 4 min S^∆^ and zero challenging behavior. His FCR averaged 0.29/min during the extensions.

#### Escape-maintained challenging behavior

Figure 2 presents results of the demands treatment for Adam’s challenging behavior (top panel) and his task cooperation (bottom panel). In FA baseline, Adam’s challenging behavior averaged 3.28 rpm, and his cooperation was 17.86%. During DRA + EXT, challenging behavior decreased by approximately 50% (*M* = 1.70 rpm), and cooperation increased to an average of 48.85%. Little difference was observed upon evaluation of the PM strategies (mean problem behavior = 1.49 rpm; mean cooperation = 36.90%). When the treatment was further modified with an enriched-break (EB) form of reinforcement, challenging behavior decreased significantly (*M* = 0.62 rpm), and cooperation increased to 100% in the final 3 sessions. Next, the effects of DRA + EXT + EB were evaluated, removing the PM strategies. During these sessions, challenging behavior remained minimal (*M* = 0.33 rpm); although Adam’s cooperation decreased (*M* = 23.65%). When PMs were reinstated with EB (i.e., DRA + EXT + PM + EB), Adam’s challenging behavior decreased again (*M* = 0.23 rpm), and his cooperation improved to an average of 76.67%. A brief treatment reversal to the initial DRA + EXT was associated with a substantial increase in challenging behavior (3.00 rpm) and zero cooperation. Adam’s final treatment package of DRA + EXT + PM + EB was then associated with a 91.16% reduction in challenging behavior (*M* = 0.29 rpm), along with significantly improved cooperation (*M* = 73.40%). Adam’s behavior reduced further in extensions and follow-up (*M* = 0.14 rpm; 95.73% reduction) despite an initial increase in two sessions. No challenging behavior was observed in his final 4 sessions. Similarly, his cooperation improved across sessions to 100%.

### Social validity

Following treatment, Adam’s mother completed The Intervention Rating Profile (IRP; [22]) a 6-point rating scale designed to measure a parent’s degree of treatment acceptability. Her total score was 88 out of 90, indicating a highly favorable rating of the treatment package. She assigned a score of 4 to an item asking about her thoughts on the intervention being appropriate for other children.

## Discussion

This report used single case experimental designs (e.g., Baer et al., 1968) to evaluate the effectiveness of behavioral, vision-related, and combined treatment components on reducing behavior problems and increasing the adaptive behavior of a child with SCN2A-RD and associated ID, ASD, and CVI. Research consistently shows that treatments guided by FBA are most effective in reducing behavior problems (e.g., [15]). Results of the present report indicate that combining components derived from FBA and FVA were most effective for this child. Results therefore highlight best-practice of a multi-disciplinary and family-centered approach to treating behavioral challenges in individuals with CVI and communication deficits (e.g., [21]). For instance, Robertson et al. [26] showed that collaboration between ABA professionals (e.g., BCBA®) and teachers of students with visual impairment can improve adoption of function-based behavior plans in the classroom. The current study extends Robertson et al. by combining efforts at the assessment phase (i.e., FVA + FBA) to create effective family-based behavioral interventions for a child with complex vision needs.

CVI is also associated with learning, communication, and adaptive behavior deficits [4]. Along these lines, Itzhak et al. (2020) suggested the importance of quality assessments to prevent misdiagnosing children with CVI as “inattentive, incapable, or unmotivated.” Adam’s CVI presentation appears to have encompassed all of these concerns. Prior to treatment, Adam’s mother reported completing most tasks for him, to such an extent that his challenging behavior was minimal at home; although excessive in other settings such as school. McKillop et al. [24] summarized parent experiences of coping and adapting strategies to support their child with CVI’s daily functioning were commonplace and often helpful (e.g., only wearing the same clothing to be identifiable, storing favorite items in specific locations). Similar accommodation approaches have been suggested to occur in families of children with severe behavior problems. However, parents may behave or arrange the environment in particular ways to *avoid or suppress* behavior problems, inadvertently reinforcing the very problems they seek to decrease (e.g., [34]). In general, parent involvement in behavioral treatment improves outcomes and family life. Adam’s mother received ongoing training to be his primary “therapist” in his assessment and treatment. Involving his mother early in the process also helped contextualize the treatments for their home (e.g., [25]). His mother established goals for Adam to wait patiently for meals while she prepared them, along with daily living skill expectations important to the family.

There are a few limitations to Adam’s treatment worth noting, along with suggestions for future research and practice. The present demands treatment selected simple tasks and emphasized prompts that manipulated task stimuli (e.g., movement, placement), rather than changing the stimulus itself (e.g., size, color, complexity). Additionally, several PM components were combined in a package. Although quite effective, it is unclear which components might be more effective for him or necessary during certain tasks. Future research and practice could address this concern through component analyses of FVA-guided treatment components, and with further evaluation of demand types and task stimuli for individuals with CVI [3].

Individuals with CVI often require communication support with an augmentative and alternative communication approach (AAC; [21]). Adam’s FCT + EXT treatment for tangible-edibles benefited from FVA recommended enhancements. The low-tech card exchange and visually simple 2 color (red/green) signaled availability format of FCT in the current study appeared to meet Adam’s needs for improving functional communication. In particular, the red/green colored board used to correlate the availability schedule of reinforcement provided salient stimuli that were noticeably different from Adam’s surroundings (e.g., furniture, caregiver clothing, items on the Table [28],). This level of contrast may have improved Adam’s figure-ground perception (e.g., [35]) which in turn increased his ability to discriminate when he would/ would not receive snacks for appropriate communication. Behaviorally, an additional advantage of using the contrived, two-color signal board was that there was reduced likelihood the stimuli were associated as environmental cues that problem behavior would be reinforced [28].

Although the package proved effective for Adam and did not require further testing, others with CVI may require systematic evaluation of which communication modalities or related stimulus properties (e.g., complexity, color, etc.) may be most efficient or preferred (e.g., [23, 36]). Finally, research strongly supports applied behavior analysis (ABA) approaches to developmental and behavioral interventions for individuals with NDDs [13, 15, 20, 37]. ABA methods, including FBA and FA, can involve ongoing data collection and single case designs that enable clinicians/educators to rapidly assess behavioral baselines and progress to personalize, evaluate, and modify interventions.

The present report illustrates a vision-informed ABA approach for an individual with CVI in the setting of SCN2A-RD. Coupling FBA with FVA-guided recommendations resulted in a precision therapy involving vision accommodations within a function-based treatment of challenging behavior (e.g., [14]). The rate of CVI is increasing in NDD populations with medical and genetic etiologies, in part due to improved methods for detection [8]. CVI requires accommodations and may also contribute to creating conditions that lead to frustration and behavioral dysregulation in children with NDDs. Therefore, vision-informed ABA warrants further exploration for these populations.

## Data Availability

No datasets were generated or analysed during the current study.
